# Diabetes and calcific aortic valve disease: implications of glucose-lowering medication as potential therapy

**DOI:** 10.3389/fphar.2025.1583267

**Published:** 2025-04-28

**Authors:** Feng Liu, Haipeng Cai

**Affiliations:** Department of Cardiology, Taizhou Central Hospital (Taizhou University Hospital), Taizhou, China

**Keywords:** calcific aortic valve disease, diabetes, glucose-lowering medication, dipeptidyl peptidase-4 inhibitors, peroxisome proliferator-activated receptor γ agonists

## Abstract

Calcific aortic valve disease (CAVD) is a progressive disease, of which the 2-year mortality is >50% for symptomatic disease. However, there are currently no pharmacotherapies to prevent the progression of CAVD unless transcatheter or surgical aortic valve replacement is performed. The prevalence of diabetes among CAVD has increased rapidly in recent decades, especially among those undergoing aortic valve replacement. Diabetes and its comorbidities, such as hypertension, hyperlipidemia, chronic kidney disease and ageing, participated jointly in the initiation and progression of CAVD, which increased the management complexity in patients with CAVD. Except from hyperglycemia, the molecular links between diabetes and CAVD included inflammation, oxidative stress and endothelial dysfunction. Traditional cardiovascular drugs like lipid-lowering agents and renin-angiotensin system blocking drugs have proven to be unsuccessful in retarding the progression of CAVD in clinical trials. In recent years, almost all kinds of glucose-lowering medications have been specifically assessed for decelerating the development of CAVD. Based on the efficacy for atherosclerotic cardiovascular disease and CAVD, this review summarized current knowledge about glucose-lowering medications as promising treatment options with the potential to retard CAVD.

## 1 Introduction

**FIGURE 1:**
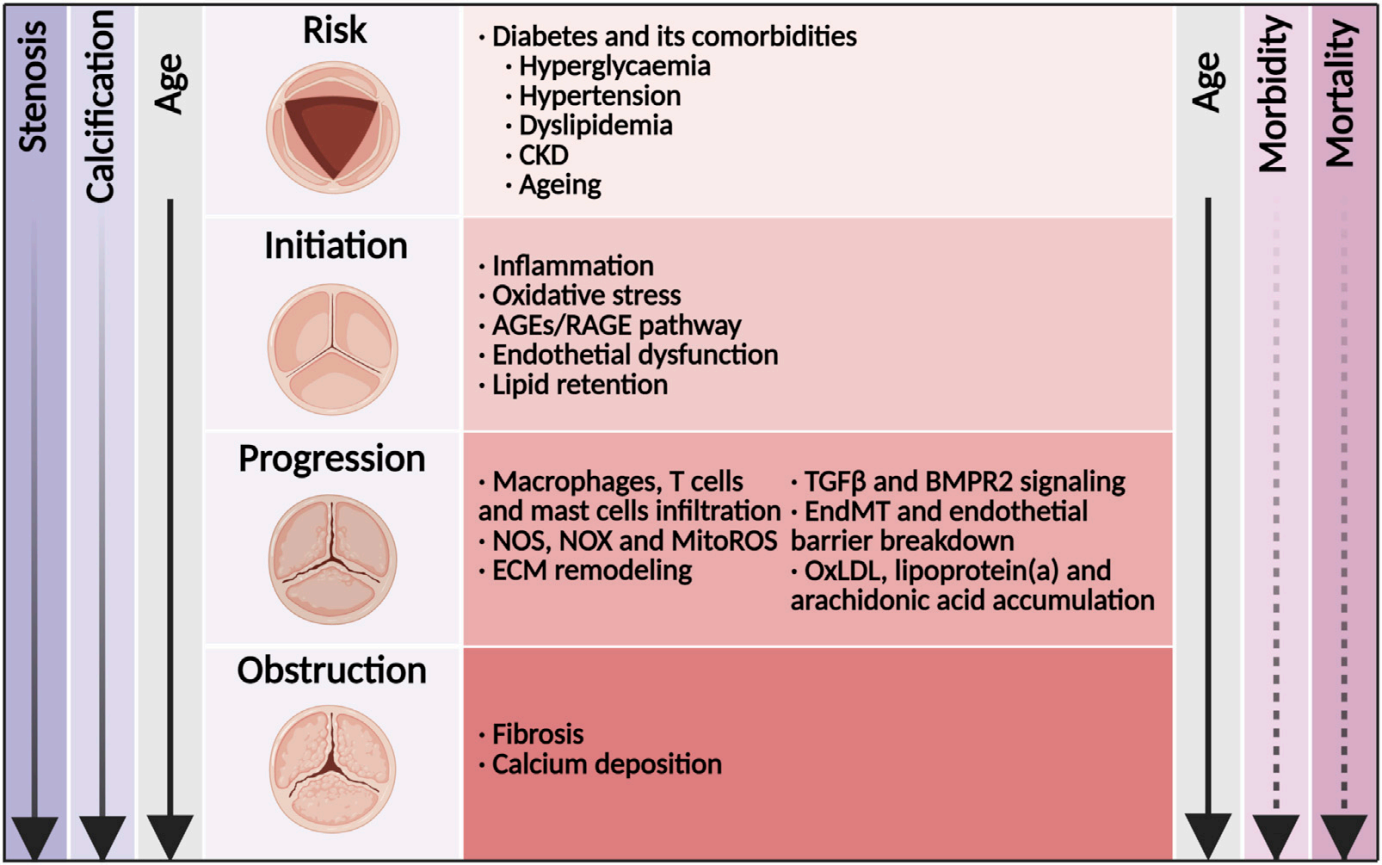
Disease mechanisms and time course of diabetes concomitant to calcific aortic valve disease. Shown was the relationship among disease stage, valve anatomy, risk factors, molecular links, and the age of the patient. With age, the morbidity of aortic valve stenosis (solid line) increased rapidly. Once in symptomatic stage, the mortality of aortic valve stenosis (dashed line) increased rapidly. CKD, chronic kidney disease; AGEs, advanced glycation end products; RAGE, receptor for AGEs; NOS, nitric oxide synthase; NOX, nicotinamide adenine dinucleotide phosphate oxidase; MitoROS, mitochondria-generated reactive oxygen species; TGFβ, transforming growth factor β; ECM, extracellular matrix; EndMT, endothelial-to-mesenchymal transition; OxLDL, oxidized low-density lipoprotein.

Calcific aortic valve disease (CAVD) is a progressive disease that is highly age-related, and its prevalence is increasing rapidly in high-income countries (Coffey et al., 2021). CAVD is characterized by thickening of the aortic valve leaflets. Disorganization and excess production of extracellular matrix (ECM) leading to fibrosis and mineralization are hallmarks of CAVD (Figure 1; Rajamannan et al., 2011). CAVD can progress over the course of years, with a substantial rise in transaortic velocities, which is referred to as aortic valve stenosis (AVS). Severe AVS is often associated with symptoms, such as shortness of breath, angina and syncope. Although mortality did not increase when AVS was asymptomatic, the 2-year mortality was more than 50% for patients with symptomatic disease unless transcatheter aortic valve replacement (TAVR) or surgical aortic valve replacement (SAVR) was performed promptly (Leon et al., 2010; Makkar et al., 2020). Global deaths from CAVD increased by 138% between 1990 and 2019 (Coffey et al., 2021). In 2017, there were an estimated 12.6 million cases of CAVD and an estimated 102,700 deaths from CAVD globally (Yadgir et al., 2020). Efforts to clarify modifiable risk factors were necessary if progress was to be made toward reducing, and eventually eliminating, the burden of this highly treatable diseases (Yadgir et al., 2020). Despite important advances in interventions to treat the terminal stage of CAVD, no pharmacotherapies are currently available to prevent or reverse disease progression. Mounting evidence has indicated that CAVD was an active and regulable pathological process in which the risk factors, such as diabetes, hypertension and hyperlipemia, were similar to those of other cardiovascular diseases (Rajamannan et al., 2011; Yan et al., 2017). However, lipid-lowering therapy with atorvastatin, simvastatin and ezetimibe, or rosuvastatin did not prevent the development of CAVD (Cowell et al., 2005; Rossebø et al., 2008; Chan et al., 2010). Therefore, the pathogenesis and molecular mechanism of CAVD need to be further explored. In this complex environment, initiation and progression of the disease thus seemed to be multifactorial, because aspects of inflammation, oxidative stress, endothelial dysfunction, lipid retention, platelet activation, ECM remodeling, cellular senescence, and biomechanical forces have all been implicated in disease development (Moncla et al., 2023; Yu Chen et al., 2023). The cellular mechanism for CAVD involved valvular endothelial cells (VECs) and valvular interstitial cells (VICs). Therein, differentiation of VICs to osteoblast- and myofibroblast-like cells led to the progressive calcification and fibrosis of aortic valve leaflets. (Rutkovskiy et al., 2017).

Patients with diabetes were at incremental risk of developing cardiovascular disease with its manifestations of coronary artery disease, stroke, peripheral artery diseases and CAVD. According to large-scaled retrospective observations worldwide, the prevalence of diabetes in CAVD ranged from 11.4% to 31.6%, and increased by almost 50% in recent decade (Taniguchi et al., 2015; Singh et al., 2017; Culler et al., 2018; López-de-Andrés et al., 2018). It was worth noting that the prevalence of diabetes in CAVD undergoing TAVR or SAVR also increased rapidly in recent years (López-de-Andrés et al., 2018; Strange et al., 2022). In addition, diabetes was a major risk factor for developing hypertension, hyperlipemia, chronic kidney disease (CKD) and ageing (Tomic et al., 2022), which in themselves were related to developing CAVD (Figure 1; Moncla et al., 2023). The combination of diabetes with these cardio-renal and metabolic comorbidities increased the risk to CAVD incidence as well as cardiovascular and all-cause death. The underlying mechanism of diabetes and its comorbidities involved oxidative stress, endothelial injury, immune cell infiltration, lipid accumulation, as well as subsequent osteoblastic/myofibroblastic differentiation of VICs and eventual calcification (Figure 1; Moncla et al., 2023; Goody et al., 2020). Once fibrosis and calcification of the aortic valve initiated, the majority of patients developed AVS progressively (Novaro et al., 2007; Owens et al., 2010).

Over the last decade, the results of numerous large cardiovascular outcome trials in patients with diabetes at high cardiovascular risk with novel glucose-lowering medications, such as sodium-glucose co-transporter-2 (SGLT-2) inhibitors and glucagon-like peptide-1 (GLP-1) receptor agonists, have substantially offered more available medications, resulting in brand new evidence-based medical therapy for the management of this population (Marx et al., 2023). Various experiments and small-scaled clinical studies have revealed the therapeutic effect of conventional anti-diabetic drugs on CAVD, such as metformin, pioglitazone and sitagliptin (En et al., 2021; Li et al., 2012; Choi et al., 2017), while the effect of new-type glucose-lowering medications on CAVD still remained unknown.

In this review, we presented the prevalence of diabetes in patients with CAVD. Then, we discussed the underlying mechanisms and molecular links between CAVD and diabetes. Most importantly, we summarized updated knowledge about the potential role of glucose-lowering medications on retarding the progression of CAVD. We made an exploration of some innovative medication options in CAVD.

## 2 The prevalence of diabetes in CAVD

With the ageing of population, the prevalence of diabetes in CAVD has increased rapidly by years (Table 1). The CURRENT AS registry, enrolling 3,815 consecutive patients with CAVD in Japan between 2003 and 2011, showed that 11.4% of CAVD had concomitant diabetes (Taniguchi et al., 2015). The multi-central, prospective, observational PRIMID AS study, conducted in 10 hospitals in United Kingdom between 2012 and 2014, showed that 14.4% of patients with moderate to severe AVS had concomitant diabetes (Singh et al., 2017). Among 180 patients with mild AVS, the peak systolic gradient across aortic valve in diabetes increased by 1.7 mmHg per year than those in non-diabetes (Aronow et al., 2001). The progression of stenosis measured by aortic valve area was significantly faster in diabetes vs non-diabetes, especially in moderate AVS cohort (Kamalesh et al., 2009). During up to 20 years of follow-up, 1.4% participants developed AVS in a prospective population-based study which enrolled 5,079 participants including 1,311 diabetes (Martinsson et al., 2014). After age- and sex-adjustments, diabetes were significantly associated with developing AVS (Martinsson et al., 2014). Diabetes had the second highest population-attributable risk of CAVD by cardiovascular risk factors, which ranked only second to hypertension (Yan et al., 2017). According to a large retrospective cohort of 2,429 consecutive patients diagnosed with CAVD, men with severe AVS more frequently had diabetes than women (Tribouilloy et al., 2021). The MESA study was the first to demonstrate that diabetes was associated with increased risk of aortic valve calcium deposits in a large, population-based, multiethnic cohort, regardless of sex (Katz et al., 2006). Although women had a lower incidence of AVS compared with men, the adjusted hazard ratios for AVS in women with diabetes did not differ significantly from those in men with diabetes (Yan et al., 2017). There were few studies illustrating the difference of type of diabetes in the occurrence of CAVD. Based on the data from two population-based prospective cohorts, enrolling 71,483 Swedish adults, type 2 diabetes mellitus (T2DM) but not type 1 diabetes mellitus (T1DM) was associated with the increased risk of CAVD (Larsson et al., 2018). Furthermore, type 2 diabetes treated with insulin was more likely to develop AVS than those only treated with oral glucose-lowering medications (Yan et al., 2017). In addition, longer duration of diabetes was associated with the increased risk of CAVD (Yan et al., 2017). In recent decades, the incidence of aortic valve replacement increased rapidly in CAVD with diabetes. A retrospective analysis of Spanish cohorts (2001–2015) showed that the prevalence of diabetes increased significantly from 16.7% to 23.5% in patients undergoing SAVR (López-de-Andrés et al., 2018). In the Danish nationwide registers, the prevalence of diabetes in patients undergoing TAVR significantly increased from 14.2% in 2008–2010 to 19.4% in 2017–2018 (Strange et al., 2022). In recent years, almost all kinds of glucose-lowering medications have been specifically assessed for decelerating the development of CAVD. Although it was quite tough that large-scale randomized controlled trials (RCTs) with long-term follow-up would be conducted to evaluate the impact of glucose-lowering medications on CAVD, optimal management of this risk factor was warranted given other established cardiovascular benefits.

**TABLE 1:** The prevalence of diabetes in CAVD.

Study type	Characteristic	Population	Conclusion	Ref.
Retrospective	CURRENT AS	3,815	11.4% of CAVD had concomitant diabetes	Taniguchi et al. (2015)
Prospective	PRIMID AS	174	14.4% of patients with moderate to severe AVS had concomitant diabetes	Singh et al. (2017)
Prospective	CANHEART	1.12 million	The prevalence of diabetes was 6% higher in AVS compared with those without AVS.	Yan et al. (2017)
Prospective		5,079	Diabetes was an independent risk factor for AVS development (HR = 3.18, 95% CI 1.51–6.69)	Martinsson et al. (2014)
Retrospective		2,429	Men with severe AVS more frequently had diabetes than women	Tribouilloy et al. (2021)
Prospective	MESA	6,780	Diabetes increased the risk of CAVD both in women (RR = 2.12, 95% CI 1.54–2.92) and in men (RR = 1.73, 95% CI 1.33–2.25)	Katz et al. (2006)
Prospective		71,483	Type 2 diabetes mellitus but not type 1 diabetes mellitus was associated with the increased risk of AVS (HR = 1.34, 95% CI 1.05–1.71)	Larsson et al. (2018)

CAVD, calcific aortic valve disease; Ref., reference; AVS, aortic valve stenosis; HR, hazard ratio; RR, relative risk; CI, confidence interval; CURRENT AS, Contemporary outcomes after sURgery and medical tREatmeNT, in patients with severe Aortic Stenosis; PRIMID AS, PRognostic Importance of MIcrovascular Dysfunction in Aortic Stenosis; CANHEART, cardiovascular health in ambulatory care research team; MESA, Multi-Ethnic Study of Atherosclerosis.

## 3 Risk factors for CAVD in diabetes

### 3.1 Hyperglycemia

Diabetes was a chronic disease characterized by hyperglycemia and frequently manifested by various cardiovascular diseases, such as myocardial infarction, stroke and CAVD. *In vitro*, elevated glucose concentration in osteogenic medium enhanced VICs matrix calcium deposits (Scatena et al., 2018). *In vivo*, diabetogenic diet fed LDLR^−/−^ApoB^100/100^ mice had higher incidence of hemodynamical AVS and valve incrassation compared with normal chow (Scatena et al., 2018). However, recent research has found that hyperglycemia-simulating environment attenuated experimentally induced calcification in cultured human VICs (Zabirnyk et al., 2024). To further mimic the pathological process of aortic valve in diabetic conditions, chronic hyperglycemia was assessed in VECs and VICs via a gelatin methacrylate 3-dimension model (Ciortan et al., 2020). The gene expression of IL-1β and MCP-1 were increased after hyperglycemic treatment for 2 weeks, exhibiting changes of inflammation and ECM remodeling (Ciortan et al., 2020). The early changes in VECs exposed to hyperglycemia were increased number of intermediary filaments and attenuation of intercellular junctions (Simionescu et al., 1996), which were related to endothelial-to-mesenchymal transition (EndMT). However, in another dynamic 3-dimension aortic valve model using a software-governed bioreactor system with controlled pulsatile flow, hyperglycemia affected insulin signaling pathways without any impact on fibrosis or calcification of aortic valve (Selig et al., 2021). This controversial relationship between hyperglycemia and CAVD indicated that there were other complicated mechanisms of diabetes on developing CAVD beyond sole hyperglycemia. The hamster developed CAVD at a much faster rate in experimental hyperlipidemia with hyperglycemia than in hyperlipidemia alone (Simionescu et al., 1996).

### 3.2 Hypertension

Hypertension was a well-known complication of diabetes and 40%–60% patients with diabetes would develop abnormal blood pressure or hypertension (Lee et al., 2017). Patients with diabetes developed increased arterial resistance caused by vascular remodeling and increased circulating volume caused by hyperinsulinemia and hyperglycemia, both of which boosted systemic blood pressure (Ohishi, 2018). Hypertension coexisted in approximately 70% patients with AVS (Linhartová et al., 2007). In a large-scaled clinical observation, enrolling 5.4 million population without known valvular heart disease, long-term exposure to elevated blood pressure was associated with an increased risk of CAVD (Rahimi et al., 2018). Specifically, each 20 mmHg increase in systolic blood pressure was associated with a 41% higher risk of CAVD (Rahimi et al., 2018). Each 10 mmHg increase in diastolic blood pressure was associated with a 24% higher risk of CAVD (Rahimi et al., 2018). Evidence existed that hypertension induced the progression of CAVD by hemodynamic flow disturbance which could cause mechanical damage to the VECs, especially on the aortic side of valve leaflet (Lindman and Otto, 2013). Valvular endothelial dysfunction was thought to enhance lymphocyte and macrophage infiltration, which aggravated ECM remodeling and calcification via secretion of various proinflammatory and procalcific cytokines (Basile et al., 2021). Angiotensin II was a key mediator in the pathophysiological process of both hypertension and CAVD. Angiotensin II was abundantly expressed in aortic valve excised from CAVD, and could induce myofibroblastic differentiation of VICs and collagen deposition (Fujisaka et al., 2013). High-dose of angiotensin II administration to ApoE^-/-^ mice promoted aortic valve incrassation, which could be suppressed by olmesartan (Fujisaka et al., 2013). However, the activity of the angiotensin-converting enzyme 2 was associated with the degree of calcification but not the severity of stenosis (Ramchand et al., 2020), indicating that further research was urgent for the role of the valvular renin-angiotensin system in CAVD pathogenesis beyond its effects on hypertension.

### 3.3 Hyperlipidemia

Hyperlipidemia was one of the major comorbidities in diabetes, which has been recognized as a hallmark in the early stage of CAVD and could be detected long before calcium deposits by PET-CT (Abdelbaky et al., 2015). A genome-wide meta-analysis of 11.6 million variants in 10 cohorts, enrolling 653,867 European ancestry participants, supported a causal contribution of lipoprotein(a) (Lp(a)), apolipoprotein B, low-density lipoprotein (LDL) to AVS (Yu Chen et al., 2023). In the Global Lipids Genetics Consortium, enrolling 188,577 participants, the odds ratio for developing AVS per unit increase in lipid parameter was 1.52 for LDL (Nazarzadeh et al., 2020), indicating that LDL-lowering medication might be effective in treating CAVD. However, three RCTs failed to illustrate any significant benefit of LDL-lowering medication with statins on the prevention of AVS (Cowell et al., 2005; Rossebø et al., 2008; Chan et al., 2010), indicating that further studies were needed to illustrate the association of other lipid indexes and CAVD. The further study of post-hoc analysis of the ASTRONOMER trial revealed that the Lp(a) was associated with faster CAVD progression (Capoulade et al., 2020). Lp(a) was a LDL-like particle synthesized by hepatocytes. As early as 1995, cross-sectional studies shown that patients with AVS exhibited higher level of Lp(a) (Gotoh et al., 1995). Recently, large-scale prospective studies found a strong dose-dependent increased risk of AVS in those with elevated level of Lp(a) (Kamstrup et al., 2014; Arsenault et al., 2014). The underlying pathological processes involving Lp(a) in CAVD included endothelial dysfunction, promotion of foam cell formation through oxidized phospholipids (OxPL), inflammation and oxidative stress (Schnitzler et al., 2020; Zheng et al., 2019). Further prospective RCTs are warranted to illustrate the effect of reduction of Lp(a) level with specific antisense oligonucleotides or short interfering RNA on retarding CAVD.

### 3.4 Chronic kidney disease

As another major complication of diabetes, approximately 30%–40% of patients with diabetes developed diabetic nephropath (Selby and Taal, 2020), which progressively caused CKD and was associated with increased cardiovascular mortality (Jin et al., 2023). The prevalence of CAVD ranged from 28% to 85% in patients with CKD (Rattazzi et al., 2013). Even in stage 2 and 3 CKD patients, >30% of the participants were found to have detectable calcification in aortic valve (Hensen et al., 2018). Moreover, functional AVS was found in 9.5% of patients with CKD compared with 3.5% of general population (Samad et al., 2017). Even when taking account of age, sex, diabetes and hypertension, patients with CKD had a 1.2- to 1.3-fold increased risk of AVS (Samad et al., 2017). Multiple mediators in patients with CKD, including hyperphosphatemia, calcium-phosphate product, parathyroid hormone, and vitamin D, have been identified as contributions to calcium deposits in aortic valve (Yutzey et al., 2014). Abnormal plasma calcium and phosphate metabolism mostly attributed to the incidence of valvular calcification in CKD patients (Maher et al., 1987). CKD concomitant low vitamin D and secondary hyperparathyroidism were related to CAVD progression (de Boer et al., 2009; Hekimian et al., 2013). Crucially, once mineralization began, alterations in the local ECM could further lead neighboring cells to adopt pathological phenotypes, resulting in a feedforward mechanism (Fisher et al., 2013). The underlying molecular mechanisms might be the activation of osteoprotegerin, RANK/RANKL axis, FGF23 and klotho in VICs (Samad et al., 2017; Rong et al., 2018). Therefore, with the deterioration of renal function and occurrence of CKD-related complications, a combination of various risk factors jointly participated in the development and progression of CAVD in patients with CKD.

### 3.5 Ageing

Ageing was a powerful independent risk factor for degenerative aortic valve disease. Diabetes was closely linked to pathological ageing and was a major risk factor for age-associated cardiovascular diseases (Beckman et al., 2002). CAVD was viewed as a degenerative process, where calcification was thought to be the consequence of physiological ageing (Rajamannan et al., 2011). Interestingly, a renewed characterization of the ageing aortic valve has started to emerge in recent years. Many prior studies that examined “healthy” elderly valves actually included valves with micro-calcificatio (Sell and Scully, 1965; Waller, 1988), meaning that those data could not be used to define the characteristics of a normal, ageing valve. That was because the majority of samples in these studies had come from individuals <60 years old, which was earlier than the age of typical CAVD onset. Thus, diabetes-related pathological ageing probably participated more in the onset and progression of CAVD compared with physiological ageing. Apart from physiologic age-related senescence, pathologic cellular senescence could be induced by cellular stressors, such as excessive mechanical stress, oxidative stress and metabolic stress (Chen et al., 2022; Song et al., 2020). Chronic excessive stress over the years resulted in the accumulation of pathologic cellular senescence in VECs, mainly on the aortic side of valve leaflet, where the blood flow was oscillatory (Fernández Esmerats et al., 2016). Deficiency in the anti-ageing klotho gene promoted high-fat-diet-induced aortic valve fibrosis in company with decreased AMPKα activity and increased RUNX2 level (Chen et al., 2016). In cultured porcine VICs, klotho-deficient serum plus cholesterol increased expression of RUNX2 and collagen I (Chen et al., 2016). Therefore, although the implication of age-related, replicative cellular senescence was not negligible, stress-induced cellular senescence played a more important role in CAVD.

### 3.6 Multifactorial interactions

Apart from individual effects, there were multifactorial interactions between those diabetic complications, which participated jointly in the initiation and progression of CAVD. Hypertension-induced hemodynamic shear stress on the aortic side induced endothelial dysfunction and hampered barrier function, which exacerbated lipid deposition in aortic valve under hyperlipidemic condition (Kraler et al., 2022). CKD caused hypertension by an interplay of factors, including water-sodium retention, renin-angiotensin system overactivation, and endothelial dysfunction (Gupta et al., 2023), which were common pathological factors of CAVD. Hyperglycemia represented a key cellular stress in the kidney by altering cellular metabolism in endothelial cells and podocytes (Mohandes et al., 2023). Whereafter, increased oxidative stress and activation of inflammatory pathways caused progressive kidney function decline and fibrosis (Mohandes et al., 2023). Hyperlipidemia was associated with low-grade systemic inflammation, which might lead to insulin resistance, insulin deficiency, and consequent hyperglycemia (Masenga et al., 2023). As two of the subsets of metabolic syndrome, hyperlipidemia and hyperglycemia jointly promoted CAVD via oxidative stress and chronic inflammation (Kraler et al., 2022).

## 4 Molecular links between CAVD and diabetes

### 4.1 Inflammation

**FIGURE 2:**
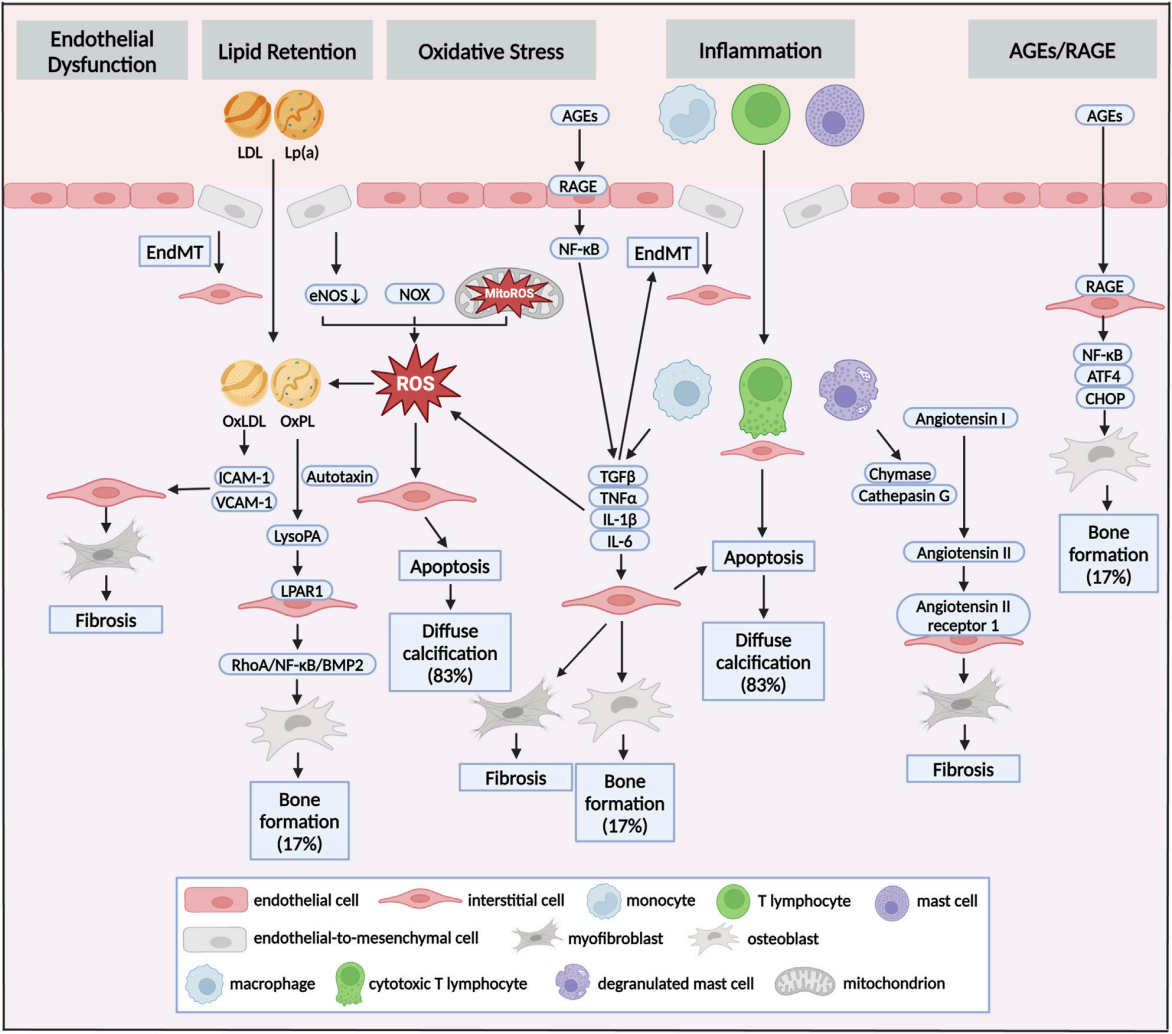
Pathways and mechanisms of diabetes concomitant to calcific aortic valve disease. Pathomechanisms of initiation and progression of CAVD in diabetes could be seen as crosstalk of distinct pathways: endothelial dysfunction, lipid retention, oxidative stress, inflammation and AGEs/RAGE. Different stimuli induced endothelial dysfunction of VECs, which allowed infiltration of lipoproteins and immune cells. This infiltration was accompanied by the production of ROS via dysregulation of eNOS, and accumulation of NOX and MitoROS. Oxidative stress could promote the transformation of LDL to OxLDL and the formation of OxPL in LP(a), which induced fibrocalcific differentiation of VICs, and eventual fibrosis and bone formation in aortic valve. Myofibrogenic and osteogenic differentiation of VICs were also stimulated by infiltrated macrophages, T lymphocytes and mast cells via secretion TGFβ, TNFα, IL-1β and IL-6. While, those cytokines aggravated EndMT in turn. Besides, cytotoxic T lymphocytes and accumulation of ROS promoted apoptosis of VICs, resulting in diffuse calcification. Increased circulating AGEs was one of the pathological features of diabetes. AGEs promoted inflammation and pro-osteogenic reprogramming via RAGE/NF-κB pathway. Diffuse calcification accounted for approximately 83% of all calcification deposits, while bone formation accounted for about 17%. LDL, low-density lipoprotein; Lp(a), lipoprotein(a); AGEs, advanced glycation end products; RAGE, receptor for AGEs; EndMT, endothelial-to-mesenchymal transition; ROS, reactive oxygen species; eNOS, endothelial nitric oxide synthase; NOX, nicotinamide adenine dinucleotide phosphate oxidase, MitoROS, mitochondria-generated ROS; OxLDL, oxidized LDL; OxPL, oxidized phospholipids.

Diabetes was associated with upregulated factors of inflammatory response, cytokines and coagulation at the initiation and propagation stage of CAVD. In 2012, Dweck MR and co-workers have assessed the calcification and inflammation in the same patient at the same time by using 18F-Flurodeoxyglucose and 18F-Sodium fluoride PET/CT (Dweck et al., 2012). They found that inflammatory response increased by 91% in CAVD compared with healthy donors (Dweck et al., 2012). By conducting single-cell RNA sequencing with excised aortic valve from patients with CAVD, VICs (72.64%) accounted for a major proportion, followed by monocytic cells (19.52%), lymphocytes (6.23%), VECs(1.28%) and mast cells (0.33%) (Lyu et al., 2023). Immunohistochemistry staining also showed chronic immune cells infiltration in calcified valve leaflets, composed by CD68^+^ macrophages, CD3^+^ T lymphocytes and mast cells (Figure 2), whilst only few unactivated immune cells were presented in healthy leaflets (Otto et al., 1994). Therefore, enrichment of activated, infiltrated immune cells in aortic valve was a critical pathological process in CAVD. Monocytes and macrophages could promote the osteogenic differentiation of VICs as well as calcification of aortic valve through release of extracellular vesicles and secretion of TNFα, IL-1β and IL-6 (Isoda et al., 2010; Kim, 1976; Proudfoot et al., 2000). These extracellular vesicles were filled with calcium-phosphate complexes and could form hydroxyapatite crystals, which came into contact with the ECM and functioned as nucleation sites for further microcalcification (Kim, 1976; Proudfoot et al., 2000). The described T lymphocytes infiltration in the aged and diseased aortic valve included both CD4^+^ helper lymphocytes and CD8^+^ cytotoxic lymphocytes, accompanied with release of interferon-γ (Coté et al., 2013). Interferon-γ specifically has been shown to impair the calcium resorption potential of osteoclasts through the receptor activator of RANK (Nagy et al., 2017). The infiltrated CD8^+^ cytotoxic lymphocytes in diseased aortic valve induced apoptosis of VICs by direct interaction (Coté et al., 2013). The bulk of mast cells have been activated to degranulate and release chymase and cathepsin G in calcified aortic valve, which both could convert angiotensin I to angiotensin II (Helske et al., 2004). Notably, exposure of VICs to angiotensin II promoted type I collagen synthesis by binding to the angiotensin II receptor 1 (Katwa et al., 1995). Accordingly, exposure of ApoE^-/-^ mice to high-dose angiotensin II contributed to myofibroblastic differentiation of VICs and eventual aortic valve leaflet thickening (Fujisaka et al., 2013).

### 4.2 Oxidative stress

Diabetes and its complications were associated with an oxidative stress state in which reactive oxygen species (ROS)-producing enzyme were activated and overexpressed, whilst the expression and activity of antioxidant mediators were downregulated (Sugamura and Keaney, 2011). Emerging evidences pointed out the pro-apoptotic role of oxidative stress in CAVD (Figure 2; Greenberg et al., 2022). In excised aortic valve from CAVD, superoxide and H_2_O_2_ levels were markedly increased near the calcified regions of the leaflets (Miller et al., 2008). Along similar lines, elevated superoxide was presented in the calcified aortic valve excised from both hypercholesterolemic LDLR^−/−^ApoB^100/100^ mice and high cholesterol-fed rabbits (Weiss et al., 2006; Liberman et al., 2008). *In vitro*, TGFβ induced ROS production in VICs, subsequently leading to osteogenic differentiation via a signaling cascade involving P38 MAPK and MEK1/2/ERK1/2 pathways (Das et al., 2013). Three major sources of ROS were uncoupled nitric oxide synthases (NOS), nicotinamide adenine dinucleotide phosphate oxidases (NOX), and mitochondria-generated ROS (MitoROS) (Forrester et al., 2018). Exogenous TNFα and H_2_O_2_ promoted uncoupling of endothelial NOS (eNOS), leading to increases in superoxide and H_2_O_2_ levels, which drove ECM remodeling and aortic valve calcification (Farrar et al., 2015). Accordingly, L-NAME, a NOS inhibitor, could reduce superoxide production by over 50% in calcified human aortic valves (Miller et al., 2008). Recent evidences have demonstrated that isoform specific NOX-derived ROS was might be involved in the development of CAVD. Intense NOX2 accumulation was found in the VICs osteogenesis and calcified regions of aortic valve (Liu et al., 2020). In hypercholesteremic mice, the mRNA level of NOX2 was increased in harvested valve leaflets, while no change was observed for NOX4 (Chu et al., 2013). Mitochondrion was an critical organelle responsible for both ROS production via end-product from oxidative phosphorylation and ROS elimination via mitochondrial superoxide dismutase-mediated dismutation of superoxide (Cadenas, 2018). In cultured human VICs, treatment with Lp(a) promoted VICs osteogenic differentiation accompanied with MitoROS production (Yu et al., 2018). Our recent study revealed that both β-glycerophosphate acid and TGFβ treatment stimulated MitoROS production in VICs, which was accompanied by decreased mitochondrial biogenesis and mitochondrial dysfunction (Liu F. et al., 2022). Loss of mitochondria was found in aortic valve excised from patients with CAVD and hypercholesterolemic LDLR^-/-^ mice (Liu F. et al., 2022).

### 4.3 Lipid retention

Abnormal lipid metabolism was one of the pivotal pathophysiologic characteristics of diabetes. There were growing evidences that lipid retention in the aortic valve initiated the osteogenic differentiation of VICs (Yu et al., 2017; Nsaibia et al., 2017). Histological analysis of excised calcified aortic valve has revealed the infiltration of several apolipoproteins (apo), such as apoB, apoE, apoA1, as well as fatty acids (O'Brien et al., 1996; Plunde and Bäck, 2021). Accumulation of ROS could promote the transformation of LDL to oxidized LDL (OxLDL) and the formation of OxPL in Lp(a), which have been proven to promote the osteogenic differentiation of VICs *in vitro* studies (Figure 2; Parhami et al., 1997; Tsimikas and Witztum, 2008). OxLDL increased the expression of cell adhesion molecules, including ICAM-1 and VCAM-1, which consequently promoted the inflammation and fibrocalcific remodeling in aortic valve (Côté et al., 2008; Que et al., 2018). Liquid chromatography–tandem mass spectrometry demonstrated that lysophosphatidic acid (LysoPA), the decomposition production of OxPL catalyzed by autotaxin, was exhibited in calcified valve leaflets (Saga et al., 2014). Immunohistochemical studies have also found the co-localization of Lp(a) and autotaxin in the region adjacent to calcification of aortic valve (Bouchareb et al., 2015). Further study has shown that OxPL and LysoPA could accelerate the osteogenic differentiation of VICs by the LysoPA receptor 1 (LPAR1), levels of which were also increased in CAVD (Torzewski et al., 2017). Inhibition of LPAR1 reduced the progression of AVS and calcium deposits in aortic valve (Nsaibia et al., 2017). LPAR1 could instigate a pro-calcific gene program via activation of RhoA/NF-κB/BMP2 pathway (Nsaibia et al., 2017). Fatty acids served as substrates for many lipid mediators, which was identified as an important mechanism in the development of CAVD. Arachidonic acid was abundant in stenotic aortic valve (Lehti et al., 2013). Phospholipase A2, responsible for making arachidonic acid available for downstream metabolism, was associated with the expression of osteogenic markers including BMP2, osteopontin and ALP (Suzuki et al., 2014). However, a higher omega-3 index was associated with echocardiographic signs of a slower progression of CAVD in retrospective analysis (Artiach et al., 2020). EPA-RvE1 was abundant in non-calcified aortic valve (Artiach et al., 2020). Deficiency of ChemR23, a receptor of EPA-RvE1, displayed aggravated aortic valve calcification in hyperlipidemic mice (Artiach and Bäck, 2020), regarding the EPA-RvE1 as a beneficial fatty acid in CAVD.

### 4.4 AGEs/RAGE pathway

Exposure to increased hyperglycemia in diabetes rapidly accelerated circulating advanced glycation end products (AGEs) formation. In a study enrolled 76 patients with severe AVS, both plasma and valvular AGEs level were associated with the severity of AVS in patients with concomitant diabetes (Kopytek et al., 2020). Receptor of AGEs (RAGE) were partially co-existed with osteocalcin and ALP in the aortic valve excised from patient with CAVD, evaluated by immunohistochemistry (Saku et al., 2020). The rabbit and mouse models of CAVD proved that AGEs accumulation within aortic valves resulted in osteogenic differentiation of VICs (Li et al., 2012; Hofmann et al., 2014). Extracellular AGEs modified global tissue structure and function through binding to RAGE, which mediated multiple cellular processes, such as inflammatory activation, endoplasmic reticulum stress and ECM remodeling (Du et al., 2018; Kopytek et al., 2021). AGEs-modified LDL promoted osteogenic differentiation of VICs in a dose-dependent manner through augmenting the expression of inflammatory cytokines, such as ICAM-1 and IL-6 (Yang et al., 2024). In hypercholesterolemic mice, RAGE deficiency attenuated AGEs accumulation, morphometric infiltration and calcium deposits in aortic valve (Hofmann et al., 2014). Further study demonstrated that RAGE deficiency alleviated aortic valve calcification through the inhibition of endoplasmic reticulum stress via NF-κB/ATF4/CHOP pathway (Figure 2; Wang B. et al., 2017). Moreover, AGEs/RAGE axis caused EndMT in early CAVD via TGFβ and BMPR2 signaling (Deng et al., 2020). Therefore, AGEs/RAGE axis participated an important role in the pathological process of CAVD via multiple mechanisms, and offered a potential target for the treatment of CAVD.

### 4.5 Endothelial dysfunction

The integrity of the endothelial layer was a key element of valvular homeostasis. The destruction of intimal integrity, characterized by endothelial dysfunction, usually occurred in the initial stage of CAVD, which was triggered by noxious substances (such as hyperglycemia, hypertension and hyperlipemia) (Shu et al., 2023). Once endothelial barrier was broken down, inflammatory cells, such as monocytes and lymphocytes, could infiltrate the valvular subendothelium (Conte et al., 2021). Then, with the activation by uptake of oxidized lipids, invading inflammatory cells began to differentiate and released inflammatory factors, chemokines, growth factors, and cathepsin, which have been confirmed to be closely associated with calcification and stenosis of aortic valve (Otto et al., 1994; Mathieu et al., 2015; Fondard et al., 2005). Decreased expression of eNOS was observed in calcified aortic valve compared with healthy leaflet (Richards et al., 2013). Accordingly, loss of eNOS aggravated aortic valve calcification in mice model of CAVD, whilst reinstating nitric oxide (NO) signaling effectively attenuated osteogenic differentiation of VICs, strongly indicating a protective role of VECs-derived NO in CAVD (El et al., 2014; Majumdar et al., 2021). Proper eNOS function and NO synthesis was dependent on its cofactor tetrahydrobiopterin. The reduction in endothelial-dependent tetrahydrobiopterin level increased peroxynitrite formation, resulting in osteogenic differentiation of VICs and aortic valve calcification (Liu Z. et al., 2022). In addition, eNOS was markedly decreased in aortic side of endothelium compared with disease-resilient ventricularis (Richards et al., 2013). Indeed, aortic side of valve leaflet was largely exposed to oscillatory shear stress, known to induce endothelial dysfunction and disturb NO secretion. Furthermore, under exposure to circulating stimulants, VECs could differentiate into mesenchymal valve progenitor cells, a precursor of VICs, in a process called EndMT (Goody et al., 2020). It was thought that a physical low rate of EndMT functioned to supply defunct VICs (Hjortnaes et al., 2015). However, a relatively higher rate of EndMT led to a destruction of the endothelial barrier, due to the loss of adherent junction (Figure 2; Timmerman et al., 2004). Pseudotime differentiation trajectory indicated EndMT in CAVD process through unbiased single-cell RNA sequencing for isolated human aortic valve leaflets (Xu et al., 2020). Lymphocyte and macrophage infiltrated into aortic valve through destroyed endothelial layer and secreted various procalcific and proinflammatory cytokines (Basile et al., 2021). TGFβ and IL-1β would in turn stimulate the EndMT of VECs (Kaden et al., 2003; Satta et al., 2002). When those pathological factors constantly existed in aortic valve, endothelial-derived VICs could differentiate into the osteoblastic phenotype (Goody et al., 2020).

### 4.6 Crosstalk between molecular mechanisms

The crosstalk between molecular mechanisms is illustrated in Figure 2. EndMT-related endothelial barrier destruction promoted lipid retention and inflammatory cells infiltration. Monocytes and macrophages exaggerated intracellular oxidative stress via release of TNFα, IL-1β and IL-6. Aggravated oxidative stress caused oxidation of LDL and Lp(a). Accumulation of AGEs induced the inflammatory response by bind to RAGE.

## 5 Potential role of glucose-lowering medications in CAVD

### 5.1 Metformin

A classic and widely accepted first-line therapeutic option for hyperglycemia in T2DM was the administration of metformin, currently considered as the “gold standard” therapy for this condition. This drug stood out not only for its glycemic control, but also because it promoted several improvements in endothelial dysfunction, insulin resistance, lipid profiles, oxidative stress and inflammation (Wang YW. et al., 2017). Since metformin came into clinical use in 1995, it was not required to undergo cardiovascular outcome trials. Up to the present, there have been no large-scaled RCTs to rigorously evaluate the safety and efficacy of metformin on cardiovascular outcomes. In meta-analyses of 13 clinical trials evaluating the cardiovascular efficacy of metformin vs placebo or active control, there was no statistically significant in any assessed cardiovascular outcomes (Table 2; Griffin et al., 2017). Studies regarding the efficacy of metformin on cardiovascular calcification were limited. The DIACART cross‐sectional cohort study, assessed below‐the‐knee calcification scores in 198 patients with T2DM but without severe CKD, showed that the association between metformin and calcification score was independent of age, gender, smoking, renal function, diabetes duration, neuropathy, hemoglobinA1c level, and inflammation (Mary et al., 2017). Another cross-sectional study, assessed coronary artery calcification in 369 patients with T2DM, showed that metformin was negatively associated with coronary artery calcification severity, which was independent of age, gender, smoking, diabetes duration, hypertension, statin prescription, and number of non-metformin glucose-lowering medications (Lu et al., 2019). Patients with aortic valve calcification and T2DM, under treatment with metformin, displayed less systemic oxidative stress compared with those without metformin, possibly due to the benefit of metformin (Corbacho-Alonso et al., 2023). *In vitro*, metformin alleviated phosphate- and TGFβ-induced osteogenic differentiation of human VICs through activating the PI3K/AKT pathway and β-catenin pathway which emerged as important regulatory axis in the pathological process of CAVD (En et al., 2021; Liu et al., 2018). Besides, metformin ameliorated rat VICs osteogenesis through the autophagy-mediated recycling of RUNX2 (Phadwal et al., 2023). Metformin was found to suppress diabetes-accelerated atherosclerosis in mice through DRP1 inhibition in aortic root (Wang Q. et al., 2017). Inhibition of DRP1 could attenuate aortic valve calcification by preventing matrix mineralization, cytoskeletal rearrangement, mitochondrial dysfunction, and reducing type 1 collagen secretion and ALP activity (Rogers et al., 2017). Thus, further animal experiments and large-scale RCTs were essential for verification of the efficacy of metformin on retarding CAVD.

**TABLE 2:** Main outcomes of atherosclerotic cardiovascular disease with glucose-lowering medications in diabetes.

Study type	Characteristic	Population	Objective	Contrast	Duration of follow-up (year)	MACE	Cardiovascular death	Stroke	Myocardial infarction	Hospitalization for heart failure or angina	Ref.
1. Metformin											
Meta-analysis	—	20,268	Metformin	Placebo or active control	—	—	0.97 (0.80–1.16)	1.04 (0.73–1.48)	0.89 (0.75–1.06)	—	Griffin et al. (2017)
2. Thiazolidinediones											
Prospective	PROactive	5,238	Pioglitazone	Placebo	2.8	0.84 (0.72–0.96)	—	0.81 (0.61–1.07)	0.83 (0.65–1.06)	—	Dormandy et al. (2005)
Retrospective	—	6,727	Pioglitazone	DPP-4 inhibitors	—	0.85 (0.73–0.99)	0.88 (0.70–1.1)	—	—	—	Lin et al. (2020)
Meta-analysis	—	14,703	Pioglitazone	Placebo or active control	—	0.74 (0.60–0.92)	—	0.81 (0.68–0.96)	0.77 (0.64–0.93)	1.33 (1.14–1.54)	de Jong et al., 2017
Meta-analysis	—	16,390	Pioglitazone	Placebo or active control	—	0.82 (0.72–0.94)	—	0.80 (0.62–1.04)	0.81 (0.64–1.02)	1.41 (1.14–1.76)	Lincoff et al. (2007)
3. Sulfonylureas											
Prospective	UKPDS	3,867	Sulfonylureas	insulin or diet control	>10	—	—	1.11 (0.81–1.51)	0.84 (0.71–1.00)	0.91 (0.54–1.52)	Author anonymous (1998)
4. Dipeptidyl Peptidase-4 Inhibitors											
Prospective	SAVOR-TIMI 53	16,492	Saxagliptin	Placebo	2.1	1.00 (0.89–1.12)	1.03 (0.87–1.22)	1.11 (0.88–1.39)	0.95 (0.80–1.12)	1.27 (1.07–1.51)	Scirica et al. (2013)
Prospective	EXAMINE	5,380	Alogliptin	Placebo	1.5	0.96 (≤1.16)	0.79 (0.60–1.04)	0.91 (0.55–1.50)	1.08 (0.88–1.33)	1.19 (0.90–1.58)	White et al., 2013
Prospective	TECOS	14,671	Sitagliptin	Placebo	3	0.98 (0.88–1.09)	1.03 (0.89–1.19)	0.97 (0.79–1.19)	0.95 (0.81–1.11)	1.00 (0.83–1.20)	Green et al. (2015)
Prospective	CARMELINA	6,979	Linagliptin	Placebo	2.2	1.02 (0.89–1.17)	0.96 (0.81–1.14)	0.91 (0.67–1.23)	1.12 (0.90–1.40)	0.90 (0.74–1.08)	Rosenstock et al. (2019a)
Prospective	CAROLINA	6,041	Linagliptin	Glimepiride	6.3	0.98 (0.84–1.14)	1.00 (0.81–1.24)	0.86 (0.66–1.12)	1.03 (0.82–1.29)	1.21 (0.92–1.59)	Rosenstock et al. (2019b)
5. Glucagon-like Peptide-1 Receptor Agonists											
Prospective	LEADER	9,340	Liraglutide	Placebo	3.8	0.87 (0.78–0.97)	0.78 (0.66–0.93)	0.86 (0.71–1.06)	0.86 (0.73–1.00)	0.87 (0.73–1.05)	Marso et al. (2016a)
Prospective	SUSTAIN-6	3,297	Semaglutide	Placebo	2.1	0.74 (0.58–0.95)	0.98 (0.65–1.48)	0.61 (0.38–0.99)	0.74 (0.51–1.08)	1.11 (0.77–1.61)	Marso et al. (2016b)
Prospective	HARMONY OUTCOMES	9,463	Albiglutide	Placebo	1.6	0.78 (0.68–0.90)	0.93 (0.73–1.19)	0.86 (0.66–1.14)	0.75 (0.61–0.90)	0.71 (0.53–0.94)	Hernandez et al. (2018)
Prospective	REWIND	9,901	Dulaglutide	Placebo	5.4	0.88 (0.79–0.99)	0.91 (0.78–1.06)	0.76 (0.62–0.94)	0.96 (0.79–1.15)	0.93 (0.77–1.12)	Gerstein et al. (2019)
Prospective	PIONEER 6	3,183	Oral semaglutide	Placebo	1.3	0.79 (0.57–1.11)	0.49 (0.27–0.92)	0.74 (0.35–1.57)	1.18 (0.73–1.90)	0.86 (0.48–1.55)	Husain et al. (2019)
6. Sodium-glucose Co-transporter-2 Inhibitors											
Prospective	EMPA-REG OUTCOME	7,020	Empagliflozin	Placebo	3.1	0.86 (0.74–0.99)	0.62 (0.49–0.77)	1.24 (0.92–1.67)	0.87 (0.70–1.09)	0.65 (0.50–0.85)	Zinman et al. (2015)
Prospective	CANVAS	10,142	Canagliflozin	Placebo	2.4	0.86 (0.75–0.97)	0.87 (0.72–1.06)	0.90 (0.71–1.15)	0.85 (0.69–1.05)	0.67 (0.52–0.87)	Neal et al. (2017)
Prospective	DECLARE-TIMI 58	17,160	Dapagliflozin	Placebo	4.2	0.93 (0.84–1.03)	0.98 (0.82–1.17)	1.01 (0.84–1.21)	0.89 (0.77–1.01)	0.73 (0.61–0.88)	Wiviott et al. (2019)
Prospective	CREDENCE	4,401	Canagliflozin	Placebo	2.6	0.80 (0.67–0.95)	0.78 (0.61–1.00)	—	—	0.61 (0.47–0.80)	Perkovic et al. (2019)
Prospective	VERTIS CV	8,246	Ertugliflozin	Placebo	3	0.99 (0.88–1.12)	0.92 (0.77–1.10)	1.00 (0.76–1.32)	1.04 (0.86–1.27)	0.70 (0.54–0.90)	Cannon et al. (2020)
Prospective	SCORED	10,584	Sotagliflozin	Placebo	1.3	0.74 (0.63–0.88)	0.90 (0.73–1.12)	0.66 (0.48–0.91)	0.68 (0.52–0.89)	0.67 (0.55–0.82)	Bhatt et al. (2021)
7. Insulin											
Prospective	ORIGIN	12,536	Insulin glargine	Standard care	6.2	1.01 (0.93–1.10)	0.98 (0.87–1.10)	0.92 (0.79–1.08)	1.09 (0.93–1.27)	1.02 (0.88–1.19)	Bosch et al. (2012)
Prospective	DEVOTE	7,637	Insulin degludec	Insulin glargine U100	1.99	0.91 (0.78–1.06)	0.96 (0.76–1.21)	0.90 (0.65–1.23)	0.85 (0.68–1.06)	0.95 (0.68–1.31)	Marso et al. (2016a)

MACE, major adverse cardiovascular events, cardiovascular death, stroke, myocardial infarction and hospitalization for heart failure or angina are presented as HR or OR or RR (95% Cl); OR, odds ratio; DPP-4, dipeptidyl peptidase-4; PROactive, PROspective pioglitAzone Clinical Trial In macroVascular Events; UKPDS, UK Prospective Diabetes Study; SAVOR-TIMI 53, Saxagliptin Assessment of Vascular Outcomes Recorded in Patients with Diabetes Mellitus-Thrombolysis in Myocardial Infarction 53; EXAMINE, Examination of Cardiovascular Outcomes with Alogliptin versus Standard of Care; TECOS, Trial Evaluating Cardiovascular Outcomes with Sitagliptin; CARMELINA, Cardiovascular and Renal Microvascular Outcome Study With Linagliptin; CAROLINA, Cardiovascular Outcome Study of Linagliptin vs. Glimepiride in Type 2 Diabetes; LEADER, Liraglutide Effect and Action in Diabetes: Evaluation of Cardiovascular Outcome Results; SUSTAIN-6, Trial to Evaluate Cardiovascular and Other Long-term Outcomes with Semaglutide in Subjects with Type 2 Diabetes; REWIND, Researching Cardiovascular Events with a Weekly Incretin in Diabetes; PIONEER, Peptide Innovation for Early Diabetes Treatment 6; EMPA-REG OUTCOME, Empagliflozin Cardiovascular Outcome Event Trial in Type 2 Diabetes Mellitus Patients-Removing Excess Glucose; CANVAS, Canagliflozin Cardiovascular Assessment Study; DECLARE-TIMI 58, Dapagliflozin Effect on Cardiovascular Events-Thrombolysis in Myocardial Infarction 58; CREDENCE, Canagliflozin and Renal Events in Diabetes with Established Nephropathy Clinical Evaluation; VERTIS CV, Evaluation of Ertugliflozin Efficacy and Safety Cardiovascular Outcomes Trial; SCORED, Effect of Sotagliflozin on Cardiovascular and Renal Events in Patients with Type 2 Diabetes and Moderate Renal Impairment Who Are at Cardiovascular Risk; ORIGIN, Outcome Reduction With Initial Glargine Intervention; DEVOTE, Trial Comparing Cardiovascular Safety of Insulin Degludec versus Insulin Glargine in Patients with Type 2 Diabetes at High Risk of Cardiovascular Events.

### 5.2 Thiazolidinediones

The peroxisome proliferator-activated receptor γ (PPARγ) was a nuclear receptor that played a central role as transcriptional regulator (Lehrke and Lazar, 2005). Activation of PPARγ by specific agonists, thiazolidinediones such as pioglitazone, has been widely used to treat diabetes by insulin-sensitizing and pancreatic β-cell preserving effects (DeFronzo et al., 2019). PROactive study, evaluating the cardiovascular outcomes of pioglitazone in patients with T2DM and atherosclerotic cardiovascular disease (ASCVD), found that pioglitazone did not significantly reduce the risk of primary composite endpoint compared with placebo over the 4-year study period, but it reduced the risk of all-cause mortality, non-fatal myocardial infarction and stroke by 16% (Table 2; Dormandy et al., 2005). Results from subsequent observational studies and meta-analyses have further supported the efficacy of pioglitazone in patients with ASCVD (Lincoff et al., 2007; de Jong et al., 2017; Lin et al., 2020). The CHICAGO study demonstrated that pioglitazone over a 72-week treatment period slowed the progression of carotid intima-media thickness compared with glimepiride in patients with T2DM (Mazzone et al., 2006). Single-cell RNA sequencing analysis found activated PPARγ pathway in CD36-positive VECs in hyperlipidemic mice, and conservation of PPARγ activation in non-calcified human aortic valves (Lee et al., 2022). Pathway analyses of transcript profiling of adult male swine valve endothelial populations identified PPARγ pathway was associated with the side-specific phenotype changes in VECs (Guerraty et al., 2010). *In vivo*, PPARγ activation using pioglitazone reduced valvular inflammation in hyperlipidemic mice (Lee et al., 2022). Besides, pioglitazone attenuated aortic valve calcification and stenosis in experimental hypercholesterolemic rabbits via downregulation of RAGE (Li et al., 2012). *In vitro*, pioglitazone inhibited RAGE-mediated osteogenic differentiation of VICs (Li et al., 2012). Interestingly, pioglitazone attenuated lipid deposition, calcification, and apoptosis in aortic valves of hypercholesterolemic LDLR^−/−^ApoB^100/100^ mice, improved aortic valve function, and exhibited preferential effects on aortic valves vs aorta (Chu et al., 2013). In addition, the increased pro-calcific genes in reversa mice fed with western-diet could be attenuated by pioglitazone, in aortic valve, but not aorta (Chu et al., 2013). Overall, thiazolidinediones were one of the promising glucose-lowering to alleviate CAVD independent of glucose control. Large-scale RCTs were essential for verification of the efficacy of thiazolidinediones on retarding CAVD.

### 5.3 Sulfonylureas

**FIGURE 3:**
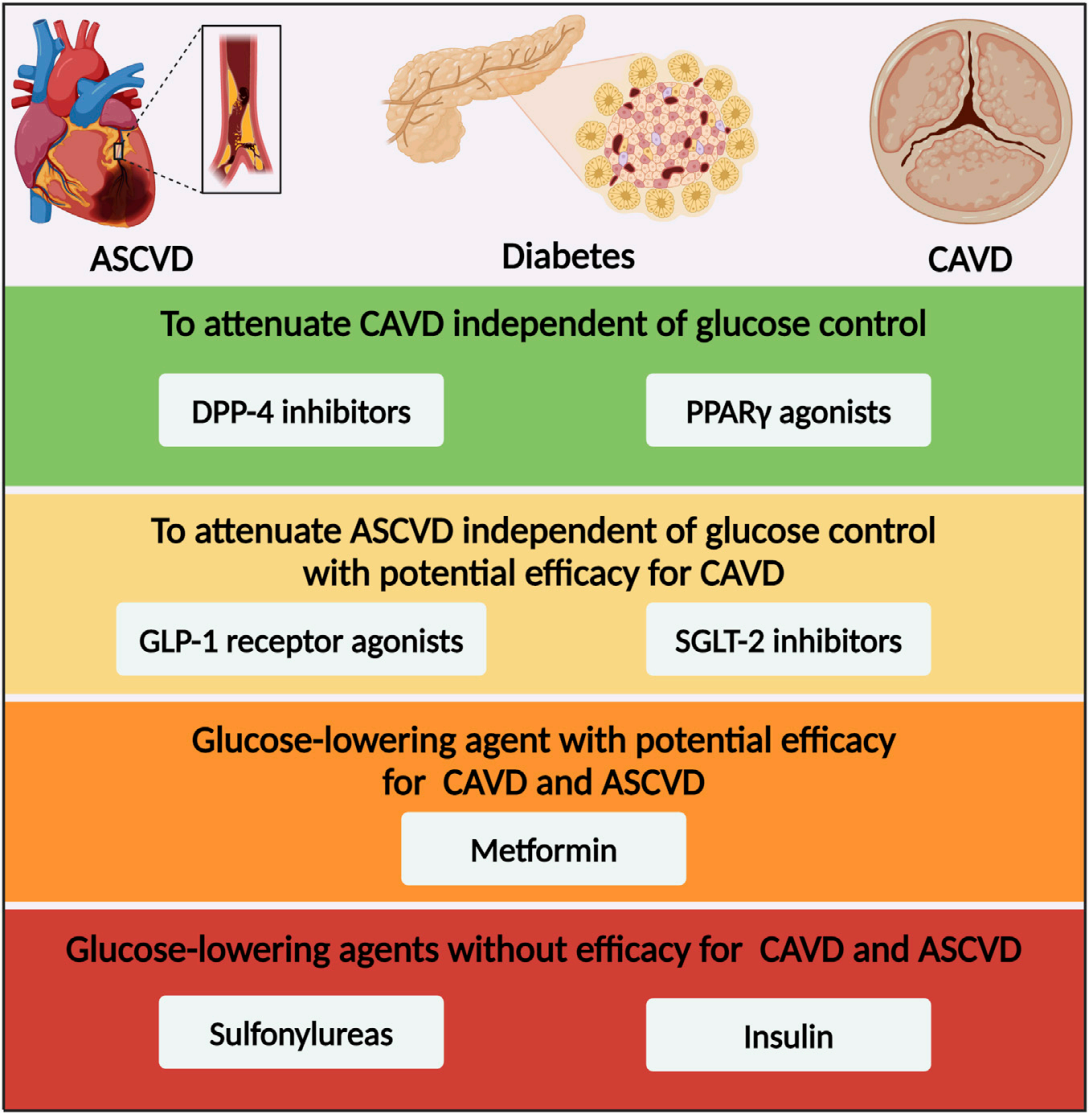
Glucose-lowering medication options for patients with diabetes and CAVD based on the efficacy for CAVD and ASCVD. ASCVD, atherosclerotic cardiovascular disease; CAVD, calcific aortic valve disease; DPP-4, dipeptidyl peptidase-4; PPARγ, peroxisome proliferator-activated receptor γ; GLP-1, glucagon-like peptide-1; SGLT-2, sodium–glucose co-transporter-2.

Since their introduction in 1950s, the sulfonylureas have remained one of the most frequently used drugs for treatment of T2DM. The sulfonylureas lowered blood glucose level by stimulating insulin release from pancreatic β cells via promoting closure of potassium channels, depolarization of cell membrane, and opening of cell-surface calcium channels (Korytkowski, 2004). In the CAROLINA trial, linagliptin was compared with the active comparator glimepiride, demonstrating no difference in any assessed cardiovascular or kidney outcomes, though noting cardiovascular safety with glimepiride (Table 2; Rosenstock et al., 2019a). Excepting glimepiride, chlorpropamide and glibenclamide did not have statistically significant effects on cardiovascular outcomes in patients with newly diagnosed T2DM, but importantly, no concerning signal of cardiovascular risk was observed (Author anonymous, 1998). Studies regarding the efficacy of sulfonylureas on cardiovascular calcification were limited and neutral. The CHICAGO trial assessed the differential effect of pioglitazone vs glimepiride on coronary artery calcium progression in T2DM (Davidson et al., 2010). The CHICAGO study demonstrated no significant difference in progression of coronary artery calcium score between pioglitazone and glimepiride (Davidson et al., 2010). The DIACART cross‐sectional cohort study, including 198 patients with T2DM, demonstrated that below-the-knee arterial calcification score was not significantly associated with the use of sulfonylureas (Mary et al., 2017). Besides, atherosclerotic plaque area and level of CD68-posivtive macrophages in aortic valve were not significantly different between glimepiride group and control group in ApoE^-/-^ mice fed with western-diet (Han et al., 2017). Overall, the current limited researches have shown neutral efficacy of sulfonylureas on cardiovascular outcomes and calcification (Figure 3).

### 5.4 Dipeptidyl peptidase‐4 inhibitors

Dipeptidyl peptidase-4 (DPP-4) inhibitors have been broadly used to treat T2DM via elevating endogenous GLP-1 level and inhibiting gastric emptying (Pratley and Salsali, 2007). DPP-4 was widely expressed in cardiovascular tissues and participated various physiopathologic roles in the process of cardiovascular diseases (Bao et al., 2014). Five RCTs in patients with T2DM at high risk of ASCVD have assessed the cardiovascular effects of DPP-4 inhibitors: saxagliptin, alogliptin, sitagliptin, and linagliptin each vs placebo, and linagliptin vs glimepiride (Table 2; Rosenstock et al., 2019a; White et al., 2013; Scirica et al., 2013; Green et al., 2015; Rosenstock et al., 2019b). All five of the trials demonstrated statistical non-inferiority but not superiority for the DPP-4 inhibitors in major adverse cardiovascular events (MACE) (Rosenstock et al., 2019a; White et al., 2013; Scirica et al., 2013; Green et al., 2015; Rosenstock et al., 2019b). The TECOS and CARMELINA trials revealed no increased risk of heart failure with either sitagliptin or linagliptin compared with placebo, indicating the cardiovascular safety of DPP-4 inhibitor (Green et al., 2015; Rosenstock et al., 2019b). Studies regarding the efficacy of DPP-4 inhibitors on cardiovascular calcification were positive and progressive. A multicenter, prospective, randomized, placebo-controlled trial, assessed the efficacy of gemigliptin on vascular calcification in diabetic kidney disease, found that the changes in the coronary artery calcium score and cardio-ankle vascular index over 6 months of the study did not significantly differ between gemigliptin and control groups (Trakarnvanich et al., 2021). However, biomarkers of vascular calcification, such as serum ALP, were decreased significantly after gemigliptin treatment (Trakarnvanich et al., 2021). Interestingly, DPP-4 inhibitors with favorable pharmacokinetic and pharmacodynamic properties were associated with lower risk of progression of AVS (Lee et al., 2020). In this study, they firstly tested the cardiac tissue distribution of five different DPP-4 inhibitors using rats and tested the anti-calcifying efficacy of five DPP-4 inhibitors using VICs (Lee et al., 2020). Then, they classified the DPP-4 inhibitors as the favorables (linagliptin and gemigliptin) and the unfavorables (alogliptin, sitagliptin and vildagliptin) in the subsequent analysis, according to the anti-calcification ability and heart/plasma ratio (Lee et al., 2020). Of note, the favorable DPP-4 inhibitors showed significantly lower maximal transaortic velocity increase than the unfavorable or non-user group, lower risk of progression of severe AVS and lower frequency of aortic valve intervention (Lee et al., 2020). *In vitro*, DPP-4 upregulation by NO deprivation-dependent NF-κB activation promoted osteogenic differentiation of VICs (Choi et al., 2017). Besides, DUSP26 enhanced DPP4 expression by antagonizing MDM2-mediated ubiquitination and degradation of DPP-4, thereby promoting CAVD progression (Wang et al., 2021). Indeed, DPP-4 inhibitors markedly reduced calcific deposition in eNOS-deficient mice and rabbit fed with cholesterol-enriched diet and vitamin D (Choi et al., 2017; Choi et al., 2021). The underlying mechanisms of DPP-4 inhibitors on retarding CAVD might be alleviating inflammation, fibrosis, and calcification (Choi et al., 2017; Lee et al., 2020; Choi et al., 2021). The application of DPP-4 inhibitor decreased the expression of inflammatory cytokines IL-2, TNFα, IL-1β, and IL-6 to suppress the progression of CAVD (Choi et al., 2021). DPP-4 inhibitor decreased the expression of osteogenic markers, such as ALP, RUNX2 and osterix, to suppress osteogenic differentiation of VICs (Choi et al., 2017; Lee et al., 2020). The use of DPP-4 inhibitor also reduced the expression of fibrosis-related genes, fibronectin 1, integrin β and collagen 1, without affecting the expression of a-SMA in VICs, indicating that DPP-4 might be involved in fibrin deposition in aortic valve (Choi et al., 2021). Overall, in order to apply DPP-4 inhibitors for treatment of CAVD, further studies were urgently needed to find the DPP4 inhibitor with higher cardiovascular tissue distribution and anti-calcification ability, and then evaluate it in large-scale RCTs.

### 5.5 Glucagon-like peptide-1 receptor agonists

GLP-1 was first identified in 1987 as one of incretins, which was released into blood by enteroendocrine L cells at a rate matched to the absorption of ingested nutrients (Holst et al., 1987). GLP-1 played numerous physiological roles by activating GLP-1 receptor, including augmented glucose-dependent insulin secretion, gastric emptying, and reduced appetite and energy intake (Tasyurek et al., 2014). Currently, GLP-1 receptor agonists were widely used in the treatment of obesity and T2DM. Beyond pancreatic islet and intestines, GLP-1 receptor was widely expressed in cardiovascular tissues, including cardiomyocytes and vascular smooth muscle cells (VSMCs) (Baggio et al., 2018; Richards et al., 2014). Emerging RCTs and real-world data, together with complementary mechanisms of action, supported the use of GLP-1 receptor agonists for the prevention of myocardial infarction, heart failure, cardiomyopathy, and atherosclerosis in patients with T2DM (Table 2; Ussher and Drucker, 2023). Semaglutide-treated patients with T2DM had a significant 26% lower risk of the primary composite outcomes of cardiovascular death, nonfatal myocardial infarction, and nonfatal stroke than those receiving placebo (Marso et al., 2016a). The LEADER trial found that liraglutide treatment decreased the incidence of MACE, cardiovascular death, and all-cause death by 13%, 22%, and 15%, in patients with T2DM at high risk of ASCVD compared with placebo (Marso et al., 2016b). Similarly, albiglutide, dulaglutide, efpeglenatide and oral semaglutide exhibited superior cardiovascular outcomes on the time to the first event of primary composite outcomes to a certain extent compared with placebo (Hernandez et al., 2018; Gerstein et al., 2019; Husain et al., 2019; Gerstein et al., 2021). Based on the cardiovascular benefits and weight-reducing effect, GLP-1 receptor agonists were recognized as the first of “Science’s 2023 Breakthrough of the Year”. At the aspect of cardiovascular calcification, some literature has revealed the association between GLP-1 and CAVD. The GLP-1 concentration in serum and calcified aortic valves excised from CAVD were both lower than those from non-CAVD (Xiao et al., 2021). *In vitro*, GLP-1 antagonized osteogenic differentiation of VICs in a dose- and time-dependent manner and it downregulated RUNX2, MSX2, BMP2, and BMP4 expression (Xiao et al., 2021). *In vivo*, plasma GLP-1 level was reduced in ApoE^-/-^ mice fed with high-cholesterol diet, and liraglutide treatment significantly attenuated calcification and inflammation in aortic valve, as well as AVS (Zhou et al., 2023). RNA sequencing and immunohistochemical data demonstrated that liraglutide treatment affected multiple pathways, involving signaling of TNFα, IL-6/JAK/STAT3, NOTCH, TGFβ, and Wnt/β-catenin (Zhou et al., 2023). However, among patients with valvular heart disease, diabetes was associated with increased fasting plasma GLP‐1 level, regardless of glucose-lowering therapy and valve phenotype, which possibly indicated a compensatory mechanism (Krizhanovskii et al., 2017). On account of the unique anti-obesity and anti-hyperglycemia dual-function, GLP-1 receptor agonists had great potential to treat CAVD with ability to attenuate ASCVD independent of glucose control (Figure 3).

### 5.6 Sodium-glucose co-transporter-2 inhibitors

SGLT-2 was mainly expressed in the proximal tubule of kidney, and was reported to be responsible for approximately 90% of renal glucose reabsorption (Kanai et al., 1994). Given the role of SGLT-2 in glucose transport, selective SGLT-2 inhibitor developed as a new class of oral anti-hyperglycemic agents available for treatment of T2DM without influence of pancreas islet function and risk of hypoglycemia (Idris and Donnelly, 2009). The first EMPAREG OUTCOME trial proposed a major therapeutic breakthrough in T2DM, that empagliflozin significantly reduced the primary composite end-points of cardiovascular mortality, non-fatal myocardial infarction and stroke by 14% (Table 2; Zinman et al., 2015). Afterwards, the results have been verified by another four cardiovascular outcome trials with SGLT-2 inhibitors and one trial of a dual SGLT-1/2 inhibitor, comprising the CANVAS program, the DECLARE-TIMI 58 trial, the CREDENCE trial, the VERTIS CV trial, and the SCORED trial (Bhatt et al., 2021; Neal et al., 2017; Wiviott et al., 2019; Perkovic et al., 2019; Cannon et al., 2020). A meta-analysis of the six RCTs with SGLT-2 inhibitors demonstrated a reduction in the primary ASCVD-based composite of time to first event of cardiovascular death, myocardial infarction, and stroke (McGuire et al., 2021). Subsequently, numerous studies have explored the underlying mechanism of SGLT-2 inhibitions on cardiovascular protective effects, such as attenuating atherosclerosis progression via inhibiting macrophage inflammation through AMPK signaling pathway (Fu et al., 2022), preventing human VSMCs proliferation and migration via targeting TRAF3IP2/ROS/NLRP3/Caspase-1-dependent IL-1β secretion (Sukhanov et al., 2021), and inhibiting formation of abdominal aortic aneurysm via p38 MAPK and NF-κB activation (Ortega et al., 2019). Given above-mentioned anti-inflammatory activity of SGLT-2 inhibitor, further experiments have verified the effects of empagliflozin on cardiovascular calcification. Empagliflozin treatment significantly alleviated atherosclerotic calcification in aortic root, and reduced the lipid level without effects on body weight and blood glucose in ApoE^-/-^ mice fed with western-diet (Li et al., 2023). *In vitro*, empagliflozin significantly inhibited osteogenic medium induced calcification of primary VSMCs and aortic rings through activating AMPK signaling pathway (Li et al., 2023). Moreover, empagliflozin attenuated vascular calcification in ApoE^−/−^mice fed with high-phosphorus diet following 5/6 nephrectomy, by regulating the NFR2/HO-1 pathway through AMPK activation (Lu et al., 2023). Empagliflozin ameliorated vascular calcification in diabetic mice through inhibiting Bhlhe40-dependent NLRP3 inflammasome activation (Li et al., 2024). It is inspiring that The New England Journal of Medicine recently published the first clinical trial designed to assess the clinical benefit and safety of the SGLT-2 inhibitor, dapagliflozin, in patients undergoing TAVR (Raposeiras-Roubin et al., 2025). Among patients with CAVD undergoing TAVR who were at high risk for heart failure events, dapagliflozin resulted in a significantly lower incidence of death from any cause or worsening of heart failure than standard care alone (Raposeiras-Roubin et al., 2025). Therefore, although the underlying mechanisms of SGLT-2 inhibitor in CAVD remained unexplored at the moment, with the advancement of experiments and clinical studies, SGLT-2 inhibitor would probably be one of the promising treatment options for CAVD with ability to attenuate ASCVD independent of glucose control (Figure 3).

### 5.7 Insulin and insulin resistance

Impaired secretion of insulin and global insulin resistance were the central pathomechanisms of diabetes. Subcutaneous insulin injection was indicated for T1DM and uncontrolled T2DM patients. Two basal insulins have been formally evaluated in dedicated cardiovascular outcome trials. In the ORIGIN trial and the DEVOTE trial, both enrolled patients with T2DM with ASCVD or at high cardiovascular risk, demonstrated no significant difference in the primary composite outcomes between insulin glargine vs standard care or insulin degludec vs insulin glargine U100 (Table 2; Bosch et al., 2012; Marso et al., 2017). The study about the association between insulin and regression of CAVD was vague. However, insulin treatment might be associated with the exacerbation of vascular calcification. Exposure to insulin glargine was associated with increased ALP activity and increased osteogenic gene expression in human VSMCs (Davenport et al., 2015). A prospective human subject study, conducted in T2DM population before and 16 months after the commencement of insulin vs oral hypoglycemics only, found that insulin treatment revealed a trend towards increased coronary artery calcification (Davenport et al., 2015). High dose of insulin treatment enhanced calcium deposits and ALP expression in β-glycerophosphate-treated human VSMCs (Olesen et al., 2007). Likewise, high fructose stimulated the osteogenic differentiation of VICs via upregulating phosphorylation of insulin receptor substrate 1 (Chang et al., 2022).

Nevertheless, insulin resistant was found as an accelerator in the progression of CAVD. The ASTRONOMER study, a multicenter randomized trial to evaluate the effects of rosuvastatin on the progression of AVS, found that metabolic syndrome was associated with progression of AVS and subsequent impairment of left ventricle geometry and function, which might be due to the insulin resistance valuated using the homeostatic assessment model index (Capoulade et al., 2012; Pagé et al., 2010). Subsequent studies confirmed that insulin resistance was a powerful independent predictor of progression to left ventricular hypertrophy in patients with AVS (Capoulade et al., 2013; Utsunomiya et al., 2014). Further study found that insulin resistance in adipose tissue but not in liver was associated with CAVD (Jorge-Galarza et al., 2016). The triglyceride-glucose index was another reliable surrogate marker of insulin resistance. The restricted cubic splines regression model revealed a linear association between the triglyceride-glucose index and the risk of all-cause mortality in patients with moderate and severe AVS (Huang et al., 2023). FABP4 and GDF-15 were associated with obesity and insulin resistance (Furuhashi et al., 2014). The patients with elevated FABP4 and GDF-15 in metabolic syndrome were at significantly higher risk of accelerated structural valve degeneration and required bioprosthetic heart valve replacement sooner (Abramov et al., 2023). The FABP4 levels were increased in the valve leaflets and VICs from patients with CAVD (Garaikoetxea et al., 2022). Osteogenic medium induced upregulation of intracellular and secreted FABP4 levels, while treatment with the specific inhibitor of FABP4 prevented increased level of inflammatory, pro-apoptotic, and osteogenic markers in VICs (Garaikoetxea et al., 2022). GDF-15 was found a significant positive association with coronary artery calcium score (Kiss et al., 2024). Genetic deletion of MMP12 ameliorated atherosclerotic plaque size and collagen deposits in aortic valve excised from LDLR^-/-^ mice fed with western-diet via improving glucose tolerance and insulin sensitivity (Amor et al., 2023). Increased protein tyrosine phosphatase 1B (PTP1B) activity was associated with cardiac insulin resistance in pressure-overloaded hearts (Nguyen et al., 2018). PTP1B inhibitors were recently considered as an attractive treatment for insulin resistance and associated metabolic disorders (Lantz et al., 2010). Our previous study has demonstrated that MSI-1436, a specific inhibitor of PTP1B, could alleviate osteogenic differentiation of VICs, calcification and stenosis of aortic valve in LDLR^-/-^ mice via improving mitochondrial dynamics (Liu F. et al., 2022). Overall, further experiments and clinical studies were needed to elucidate whether insulin accelerated the progression of CAVD. Insulin resistance might present a promising therapeutic target in patients with CAVD.

### 5.8 Limitation

There were several limitations of the existing studies between glucose-lowering medications and CAVD. First, apart from GLP-1 receptor agonists and SGLT-2 inhibitors, there were no significant cardiovascular benefits in other glucose-lowering medications. Second, although PPARγ agonists and DPP-4 inhibitors were found to attenuate CAVD independent of glucose control *in vitro* and vivo, there was lack of large-scale RCTs to further evaluate their efficacy on patients with CAVD. Moreover, although SGLT-2 inhibitor was the only glucose-lowering medication which was proved to the benefit of patients undergoing TAVR with CAVD, further experiments are warranted to demonstrate the underlying mechanisms. Lastly, it is inspiring that preliminary experiments have revealed the efficacy of GLP-1 receptor agonists on attenuating CAVD. However, more evidence of pivotal mechanisms and clinical trials are thus to advance forward.

## 6 Conclusion

In recent decades, the prevalence of diabetes has increased rapidly in patients with CAVD, especially in those undergoing TAVR or SAVR. Apart from hyperglycemia, diabetes accelerated the development and progression of aortic valve calcification and stenosis via its various comorbidities, including hypertension, hyperlipidemia, CKD and ageing, which were common risk factors of ASCVD. Except from hyperglycemia, the molecular links between diabetes and CAVD included inflammation, oxidative stress, lipid retention, AGEs/RAGE pathway activation and endothelial dysfunction. However, traditional cardiovascular drugs like lipid-lowering agents and renin-angiotensin system blocking drugs have proven to be unsuccessful in retarding the progression of CAVD in clinical trials, indicating for seeking for a more pivotal mechanism in the pathological process of CAVD. It is inspiring that the first clinical trial, designed to assess the clinical benefit and safety of the SGLT-2 inhibitor, revealed the cardiovascular benefits of dapagliflozin in patients with CAVD undergoing TAVR. Over the years, almost all kinds of glucose-lowering medications have been tested in experiments or clinical studies for sake of treating CAVD. Thereinto, overexpression of DPP-4 and inhibition of PPARγ in aortic valve were found as two of the important molecular mechanisms in the progression of CAVD. PPARγ agonists and DPP-4 inhibitors might have the efficacy to alleviate CAVD independent of their glucose-lowering effects (Figure 3). On account of the superior effects on cardiovascular outcomes, GLP-1 receptor agonists and SGLT-2 inhibitors also gained much attention in aspect of retarding CAVD. Based on the anti-obesity and anti-diabetes dual-function, GLP-1 receptor agonist was a promising medication option for CAVD. Based on the cardio-renal protective effect, SGLT-2 inhibitor was another promising medication option for CAVD. Further large-scale RCTs were essential for verification of the efficacy of glucose-lowering medications on retarding CAVD. In this regard, we summarized current knowledge about the pathophysiological mechanisms that led to diabetes concomitant to CAVD. Additionally, we highlighted some glucose-lowering medications as promising treatment options with the potential to retard CAVD.
